# Chemical Composition and Antifungal Activity of Cuminum cyminum L. Essential Oil From Alborz Mountain Against Aspergillus species

**Published:** 2012-05-28

**Authors:** Hossein Mohammadpour, Eskandar Moghimipour, Iraj Rasooli, Mohammad Hadi Fakoor, Shakiba Alipoor Astaneh, Sara Shehni Moosaie, Zeynab Jalili

**Affiliations:** 1 Department of Microbiology, Masjed Soleiman Branch, Islamic Azad University, Masjed Soleiman, IR Iran; 2 Medicinal Plant Research Center, Ahvaz Jundishapur University of Medical Sciences, Ahvaz, IR Iran; 3 Department of Biology, Shahed University, Tehran, IR Iran; 4 Department of Microbiology, Hidaj Branch, Islamic Azad University, Hidaj, IR Iran

**Keywords:** Oils, Cminum cyminum L., Antifungal Agents, Aspergillus spp.

## Abstract

**Background:**

Aflatoxin B_1_ (AFB_1_) is a highly toxic and hepatocarcinogenic metabolite produced by Aspergillus species. Some natural products are known to kill fungi and destroy toxins and toxin-producing agents.

**Objectives:**

The purpose of this study is to provide experimental data on the antifungal activity of cumin oils and their components that could be considered suitable for application in foods and drugs.

**Materials and Methods:**

The essential oil (EO) of Cuminum cyminum L. collected from Alborz Mountain, Iran, was obtained by hydro-distillation. The oil was analyzed by gas chromatography (GC) and chromatography/mass spectrophotometry (GC/MS). The antifungal activity of the oil was studied with regard to the inhibition of the growth of *Aspergillus flavus* PICC-AF39 , *Aspergillus flavus* PICC-AF24, *Aspergillus parasiticus* NRRL-2999 and Aspergillus niger. The minimal inhibitory (MIC) and minimal fungicidal (MFC) concentrations of the oil were determined.

**Results:**

α–Pinene (29.2%), limonene (21.7%), 1,8-cineole (18.1%), linalool (10.5%), linalyl acetate (4.8%), and α-terpineole (3.17%) were the major components of the essential oil from *C. cyminum* L., and the oil showed a strong inhibitory effect on fungal growth.

**Conclusions:**

Essential oils could be safely used as preservatives in pharmaceuticals as well as health and food products to protect them against toxigenic fungal infections.

## 1. Background

*Aspergillus flavus, Aspergillus parasiticus*, and *Aspergillus nomius* are able to produce aflatoxins. A. flavus produces only B aflatoxins. Aflatoxin B_1_ (AFB_1_) is the most potent carcinogen known in mammals, the risk assessment of which is well established (1, 2). Medicinal plants and spices have been used for generations by humans as food condiments and also to treat ailments because they are less toxic and generally free from side effects. *Cuminum cyminum*, which is a popular spice that is used as a flavoring agent, is widely used in food (3). The cumin seeds *Cuminum cyminum* L. belong to the family Apiaceae and are consumed in large quantities by Indians. Cumin is widely used in medicine for the treatment of dyspepsia, diarrhea, and jaundice, as it has stomachic, diuretic, carminative, and antispasmodic properties (4). China is an important exporter of this commodity and also uses it in traditional medicine. The use of natural antimicrobial compounds is important in the control of human and plant diseases of microbial origin (5). Many natural compounds have been identified that have antimicrobial activity (6-8), including essential oils (9).

## 2. Objectives

In this study, we identified the chemical composition and evaluated the antifungal properties of the essential oil of *Cuminum cyminum* L. from Alborz Mountain.

## 3. Materials and Methods

### 3.1. Plant Material and Oil Extraction

The plant (*Cuminum cyminum* L.) was collected from the Alborz mountain of Iran during June 2010. The seeds of the plant were used. The plant materials were hydro-distilled for 90 min in a full glass apparatus. The oils were extracted using a Clevenger-type apparatus. The extraction was performed after a 4-hour maceration in 500 ml of water. The oils were stored in dark glass bottles in a freezer at −12 °C until they were used. The oil yield was approximately 1%.

### 3.2. Oil Analysis

GC analyses were performed using a Shimadzu-9A gas chromatograph equipped with a flame ionization detector, and quantitative analysis was performed using Euro Chrom 2000 software from Knauer via the area normalization method, neglecting response factors. The analysis was performed using a DB-5 fused-silica column (30 m×0.25 mm, film thickness 0.25 μm, J & W Scientific Inc., Rancho Cordova, CA, USA). The operating conditions were as follows: injector and detector temperature, 250 °C and 265 °C, respectively; carrier gas, helium. The oven temperature program was 40 °C–250 °C at a rate of 4 °C/ min. The GC/MS unit consisted of a Varian Model 3400 gas chromatograph coupled to a Saturn II ion trap detector. The column was the same as in GC, and the GC conditions were as described above. The mass spectrometer conditions were: ionization potential 70 eV; electron multiplier energy 2000 V. The identities of the oil components were established from their GC retention indices, relative to C7–C25 n-alkanes, by comparison of their MS spectra with those reported in the literature (10, 11), and by computer matching with the Wiley 5 mass spectra library, whenever possible, upon co-injection with standards available in the laboratories.

### 3.3. Fungal Strains and Cultures

The microorganisms used were *Aspergillus flavus* PICC-AF39, *Aspergillus flavus* PICC-AF24, *Aspergillus parasiticus* NRRL-2999, and *Aspergillus niger*. All of these microorganisms are highly toxic and were maintained on Sabouraud Dextrose Agar (Merck, Germany) at 4 °C. Spore suspensions were prepared and diluted in sterile yeast extract sucrose (YES) broth to a concentration of approximately 106 spores/ml. The spores were counted using a hemocytometer. Subsequent dilutions were made from the above suspension and then used in the tests.

### 3.4. Antifungal Analysis

The minimal inhibitory concentration (MIC), minimal fungicidal (MFC) concentration, and fungicidal kinetics of the oils were determined. The disc diffusion method was used for antifungal screening as follows: Sterile Sabouraud Dextrose Agar (Merck) was inoculated with *Aspergillus flavus* PICC-AF39 , *Aspergillus flavus* PICC-AF24 , *Aspergillus parasiticus* NRRL-2999, and *Aspergillus niger* spores (10^6^ spores/ml) and distributed into petri plates with a diameter of 80 mm. Sterile 6-mm Whatman No. 1 filter paper was used as the disc. Under aseptic conditions, the discs were placed on the agar plates and then 5 μl, 10 μl, and 20 μl of each of the oils was put on the discs. The plates were incubated at 28 ± 2 °C for 20 days. Three replicates were used for each treatment. The diameter of the microbial inhibition zones was measured using vernier calipers. The percent mycelia inhibition was calculated by the equation: I = 1−*T/C* × 100, where I is the inhibition (%), C is the colony diameter of mycelium from a control petri plate (mm) and T is the colony diameter of mycelium from a test petri plate (mm) (11). The MIC and MFC (12) were determined by the broth dilution method in test tubes as follows: Various concentrations of the oils were added to 5 ml of YES broth tubes containing 106 spores/ml. The tubes were then incubated on an incubator shaker to evenly disperse the oil throughout the broth in the tubes. The highest dilution (lowest concentration) that showed no visible growth was regarded as the MIC. Cells from the tubes showing no growth were subcultured on Sabouraud Dextrose Agar plates to determine whether the inhibition was reversible or permanent. The MFC was determined as the highest dilution (lowest concentration) at which no growth occurred on the plates.

### 3.5. Fungicidal Kinetics of *Cuminum cyminum* L.

*Cuminum cyminum* oil from Alborz Mountain at the MFC dilution was added to 5 ml of spore suspension tubes containing 10*6* spores/ml in triplicate and then incubated at 28 ± 2 °C in an incubator shaker. Samples taken after 0, 4, 8, 12, 16, 20, and 24 hours were cultured on Sabouraud Dextrose Agar plates for 48 h at 28 ± 2 °C. The control tubes had no essential oil added. Fungal colonies were counted after incubation period, and the total number of viable spores per ml was calculated (13).

## 4. Results

**Figure 1:**
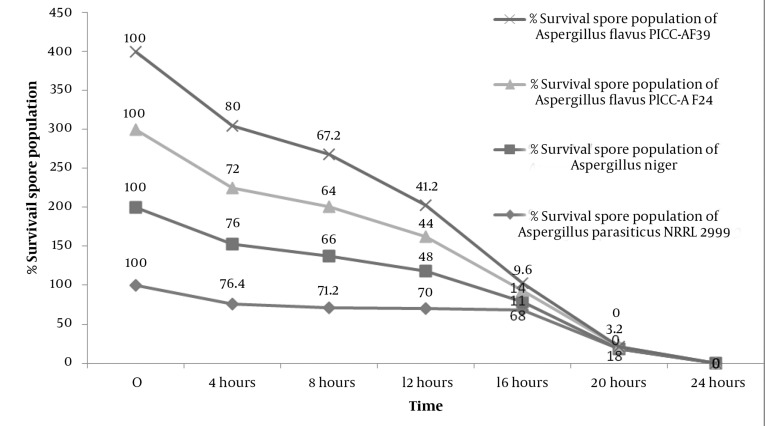
Kinetics of *Aspergillus* Spore Destruction at Minimal Fungicidal Concentrations (MFC) of the Fresh Essential Oil From *Cuminum cyminum* L. Initial Spore Concentration: 106/ml.

The results obtained by the GC and GC–MS analyses of the essential oils of *C. cyminum* L. from the Alborz Mountain range of Iran are presented in Table 1. Twenty-eight compounds were identified in the essential oil of *C. cyminum* L from Alborz Mountain. GC and GC–MS analyses revealed that *C. cyminum* L. from Alborz mountain contained α-pinene (29.2%), limonene (21.7), 1,8-cineole (18.1%), linalool (10.5%), and α-terpineole (3.17) as the major compounds. The oil yield was approximately 1%. The antifungal activities of the *C. cyminum* L. essential oil against *Aspergillus flavus* PICC-AF39, *Aspergillus flavus* PICC-AF24, *Aspergillus parasiticus* NRRL-2999, and *Aspergillus niger* were qualitatively and quantitatively assessed according to the inhibition zone diameter as well as the MIC and MFC values (Tables 2 and 3). The essential oil had substantial antifungal activity against the four fungal species tested. The four fungi species exhibited high diameters of growth inhibition (35, 55, 37 and 38 mm). At a dose of 10 μl of the oil per disc per petri plate, the *C. cyminum* L. essential oil was particularly effective against *Aspergillus parasiticus* NRRL-2999, *Aspergillus niger*, *Aspergillus flavus* PICC-AF24, and *Aspergillus flavus* PICC-AF39 with diameters of inhibition of approximately 25, 35, 23 and 23 mm, respectively, on the second day of incubation. At the end of thirtieth day, the diameter of inhibition was approximately 13, 9, 12, and 13 mm for *Aspergillus parasiticus* NRRL-2999, *Aspergillus niger*, *Aspergillus flavus* PICC-AF24, and *Aspergillus flavus* PICC-AF39 respectively. With a dose of 20 μl of the oil per disc per petri plate, the diameter of inhibition for *Aspergillus parasiticus* NRRL-2999, *Aspergillus niger*, *Aspergillus flavus* PICC-AF24, and *Aspergillus flavus* PICC-AF39 was 12, 17, 17, and 14 mm, respectively (Table 2). The antifungal activity was expressed as the MIC and MBC values (Table 3). The *C. cyminum* L. oil killed more than 60% of the spores of the four Aspergillus species tested within 12 hours. In Aspergillus flavus PICC-AF39 and *Aspergillus niger*, 100% lethality was observed within 20 hours of the exposure to the oil, whereas 100% of *Aspergillus flavus* PICC-AF24 and *Aspergillus parasiticus* NRRL-2999 spores died after 24 hours. Figure 1 shows that the spore death occurred in the first 24 hours.

**Table 1:** Chemical Composition of *Cuminum cyminum* L. Essential Oil From Alborz Mountain of Iran

No.	Alborz mountain	RI^a^	%^b^
1	Isobutyl isobutyrate	892	0.8
2	α-Thujene	922	0.3
3	α-Pinene	931	29.2
4	Sabinene	971	0.6
5	Myrcene	981	0.2
6	p-Cymene	1013	0.3
7	Limonene	1025	21.7
8	1, 8-Cineole	1028	18.1
9	γ-Terpinene	1051	0.6
10	Terpinolene	1082	0.3
11	Linalool	1089	10.5
12	α -Campholenal	1122	0.03
13	trans-Pinocarveole	1130	0.07
14	δ-Terpineole	1154	0.09
15	Terpinene-4-ol	1169	0.5
16	α-Terpineole	1180	3.17
17	trans-Carveole	1213	0.4
18	cis-Carveole	1217	0.07
19	Geraniol	1242	1.1
20	Linalyl acetate	1248	4.8
21	Methyl geranate	1310	0.2
22	α-Terpinyl acetate	1342	1.3
23	Neryl acetate	1351	0.09
24	Methyl eugenol	1369	1.6
25	β-Caryophyllene	1430	0.2
26	Spathulenol	1562	0.07
27	Humulene epoxide II	1608	0.08
28	Acetocyclohexane dione (2)	1704	0.4

^a^Retention Index Relative to n-alkane Series on the DB-5 Column.

^b^Percentage Composition of the C. cyminum L. Essential Oil.

**Table 2:** Antifungal Effects of *Cuminum cyminum* L. Essential Oil From the Alborz Mountain of Iran on *Aspergillus Spores* (106 spores/mL).

Essential oil concentration	Mold	Zone	Days of incubation																			
			2	3	4	5	6	7	8	9	10	11	12	13	14	15	16	17	18	19	20	30
(5 μl/disc)	*A.parasiticus* NRRL-2999	(mm^b^)	13	13	12	12	11	-a	-a	-a	-a	-a	-a	-a	-a	-a	-a	-a	-a	-a	-a	-a
		( %^c^ )	16.25	16.25	15.00	15.00	13.75	0	0	0	0	0	0	0	0	0	0	0	0	0	0	0
	*A.niger*	(mm^b^)	22	19	15	13	11	-a	-a	-a	-a	-a	-a	-a	-a	-a	-a	-a	-a	-a	-a	-a
		( %^c^ )	27.50	23.75	18.75	16.25	13.75	0	0	0	0	0	0	0	0	0	0	0	0	0	0	0
	*A.flavus* PICC-AF24	(mm^b^)	15	14	12	-a	-a	-a	-a	-a	-a	-a	-a	-a	-a	-a	-a	-a	-a	-a	-a	-a
		( %^c^ )	18.75	17.50	1500	0	0	0	0	0	0	0	0	0	0	0	0	0	0	0	0	0
	*A.flavus* PICC-AF39	(mm^b^)	15	13	12	12	11	11	11	11	11	11	11	11	11	11	11	11	11	11	11	11
		( %^c^ )	18.75	16.25	15.00	15.00	13.75	13.75	13.75	13.75	13.75	13.75	13.75	13.75	13.75	13.75	13.75	13.75	13.75	13.75	13.75	13.75
(10 μl/disc)	*A.parasiticus* NRRL-2999	(mm^b^)	25	23	18	15	13	13	13	13	13	13	13	13	13	13	13	13	13	13	13	13
		( %^c^ )	31.25	28.75	22.5	18.75	16.25	16.25	16.25	16.25	16.25	16.25	16.25	16.25	16.25	16.25	16.25	16.25	16.25	16.25	16.25	16.25
	*A.niger*	(mm^b^)	35	30	22	16	10	9	9	9	9	9	9	9	9	9	9	9	9	9	9	9
		( %^c^ )	43.75	37.50	27.50	20.0	12.50	11.25	11.25	11.25	11.25	11.25	11.25	11.25	11.25	11.25	11.25	11.25	11.25	11.25	11.25	11.25
	*A.flavus* PICC-AF24	(mm^b^)	23	17	15	13	13	13	13	13	13	13	13	13	12	12	12	12	12	12	12	12
		( %^c^ )	28.75	21.25	18.75	16.25	16.25	16.25	16.25	16.25	16.25	16.25	16.25	16.25	15.00	15.00	15.00	15.00	15.00	15.00	15.00	15.00
	*A.flavus* PICC-AF39	(mm^b^)	23	20	18	17	15	14	14	14	13	13	13	13	13	13	13	13	13	13	13	13
		( %^c^ )	28.75	25.00	22.50	21.25	18.75	17.50	17.50	17.50	16.25	16.25	16.25	16.25	16.25	16.25	16.25	16.25	16.25	16.25	16.25	16.25
(20 μl/disc)	*A.parasiticus* NRRL-2999	(mm^b^)	35	32	28	24	20	17	15	15	15	14	14	13	13	13	12	12	12	12	12	12
		( %^c^ )	43.75	40.00	35.00	30.00	25.00	21.25	18.75	18.75	18.75	17.50	17.50	16.25	16.25	16.25	15.00	15.00	15.00	15.00	15.00	15.00
	*A.niger*	(mm^b^)	55	46	41	38	35	28	23	21	18	18	18	17	17	17	17	17	17	17	17	17
		( %^c^ )	68.75	57.50	51.25	47.50	43.75	35.00	28.75	26.25	22.50	22.50	22.50	21.25	21.25	21.25	21.25	21.25	21.25	21.25	21.25	21.25
	*A.flavus* PICC-AF24	(mm^b^)	37	35	28	20	18	18	18	18	17	17	17	17	17	17	17	17	17	17	17	17
		( %^c^ )	46.25	43.75	35.00	25.00	22.50	22.50	22.50	22.50	21.25	21.25	21.25	21.25	21.25	21.25	21.25	21.25	21.25	21.25	21.25	21.25
	*A.flavus* PICC-AF39	(mm^b^)	38	30	25	20	20	18	18	18	15	15	15	15	15	14	14	14	14	14	14	14
		( %^c^ )	47.50	37.50	31.25	25.00	25.00	22.50	22.50	22.50	18.75	18.75	18.75	18.75	18.75	17.50	17.50	17.50	17.50	17.50	17.50	17.50

^a^Resistance

^b^Average Diameter of Fungal Inhibition Zones in Millimeters.

^c^The Percent Inhibition Occurring in 80-mm Petri Plates.

**Table 3:** Minimal Inhibitory Concentration (MIC) and Minimal Fungicidal Concentration (MFC) (ppm) of the *Cuminum cyminum* L. Essential Oil From the Alborz Mountain of Iran Against *Aspergillus Species* Spores (106 spores/ml).

Mold	MIC a	MFC a
*Aspergillus parasiticus* NRRL-2999	750 ppm	3000 ppm
*Aspergillus niger* isolated from food sample	1000 ppm	3000 ppm
*Aspergillus flavus* PICC-AF24	1000 ppm	3000 ppm
*Aspergillus flavus* PICC-AF39	1000 ppm	2500 ppm

^a^Abbreviations: MIC: Minimal Inhibitory Concentration; MFC: Minimal Fungicidal Concentration

## 5. Discussion

The main components of essential oils are reported to be different. Different combinations of *C. cyminum* L. have been reported previously (14-18).

The main oil components were cumin-alcohol (24.4%), 2-caren-10-al (20.9%), cuminal (18.4), and γ-terpinen (10.6) for *C. cyminum* from China (18). The major components reported by Lu Wang *et al*. (2009) included cuminal, cumin-alcohol, p-cymene, β-pinene, 2-care-10-al, and γ-terpinene at 22.76%, 21.76%, 14.33%, 11.06%, 8.56%, and 7.05%, respectively (16). In addition, the three major compounds obtained by Ziming Wang et al. (2006) in China were cuminal (45.75%), α-proyl-benzenemethanol (14.76%), and 2-ethylidene-3,5-heptadienal (12.91) (17). In the essential oil characterized by Rebey *et al*. (2012) from Tunisia, the major compounds were γ-terpinen (25.58%), 1-phenyl-1,2 ethanediol (23.16%), cuminaldheyde (15.31%), β-pinene (15.16%), and p-cymene (9.05%)(15). Nineteen compounds were identified in the essential oil of *C. cyminum* L. from Mashhad, Iran (Oroojalian *et al*. 2010), and the main components were cuminaldehyde (30.2%), p-cymene (14.1%), γ-terpinene (12.8%), safranal (9.4%), and β-pinene (6.4%) (19). Our analysis of *C. cyminum* L. from the Alborz mountain identified α-pinene (29.2%), limonene (21.7%), 1, 8-cineole (18.1%), linalool (10.5%), and α-terpineole (3.17%) as the major compounds. Twenty-eight compounds were identified in the essential oil of *C. cyminum* L.

In all cases, hydro-distillation was applied to extract the essential oil from *C. cyminum* L., and the extracts were analyzed by GC and GC/MS. Cumin oil possesses various compounds at different concentrations in different regions. These differences in the chemical composition of the oils could be attributed to many factors, including plant part, harvest time, extraction method, type of cultivar, storage conditions, climatic effects on the plants, and geographic origin (14-19). The oil yield in the present study was approximately 1%. The essential oil yield of cumin seeds from a local market in India was reported to be 2.33% (20). *C. cyminum* L. from the Alborz mountain had good antifungal activity against the four fungi species tested. This study indicates that *C. cyminum* L. has considerable anti-Aspergillus activity and thus deserves further investigation for clinical applications. Antimicrobial activity of *C. cyminum* L. has been reported against Gram-positive and Gram-negative bacterial species as well as yeast strains (19, 21, 22). The antibacterial effects of the EOs were assessed for several food-borne pathogens, namely *Staphylococcus aureus, Bacillus cereus, Escherichia coli* O157:H7, *Salmonella enteritidis*, and *Listeria monocytogenes*. The MIC and MBC of the essential oil were within the range of 0.37 to 3 mg/ml for *C. Cyminum* L. (19) Iacobellis, Cantore, Capasso, and Senatore (2005) reported moderate antibacterial activity of *C. cyminum* L. against some bacterial plant pathogens (22). Although the antifungal activity of *C. Cyminum* is obvious, the mechanism of action remains unclear. Further research is recommended to identify the mechanisms.
